# Potential benefits of hybrid closed‐loop systems for managing moderate‐ to high‐risk individuals with type 1 diabetes during Ramadan fasting

**DOI:** 10.1111/dom.16573

**Published:** 2025-07-07

**Authors:** Abdullah M. Alguwaihes, Metib S. Alotaibi, Hamid Alqumaidi, Mohammed A. Batais, Abdullah A. Alrasheed, Layla N. Alnasser, Janaka Karalliedde, Sufyan Hussain

**Affiliations:** ^1^ Endocrinology Unit, Internal Medicine Department College of Medicine, King Saud University Riyadh Saudi Arabia; ^2^ Diabetes Center Dallah Hospital Riyadh Saudi Arabia; ^3^ University Diabetes Center King Saud University Medical City, King Saud University Riyadh Saudi Arabia; ^4^ Department of Family and Community Medicine College of Medicine, King Saud University Riyadh Saudi Arabia; ^5^ Royal Commission Hospital/Health Services Program in Jubail (RCHSP‐J) Jubail Saudi Arabia; ^6^ Department of Diabetes, School of Cardiovascular Metabolic Medicine and Sciences, King's College London; ^7^ Department of Diabetes and Endocrinology Guy's & St Thomas' NHS Foundation Trust London UK; ^8^ Institute of Diabetes, Endocrinology and Obesity, King's Health Partners London UK

**Keywords:** automated insulin delivery, fasting, glycaemia risk index, hybrid closed‐loop systems, Ramadan, type 1 diabetes

## Abstract

**Aim:**

We evaluated whether using hybrid closed‐loop systems (HCL) provides a safer, more effective fasting experience compared with multiple daily injections with continuous glucose monitoring (MDI + CGM) for people with T1D at moderate‐to‐high risk in a real‐world setting.

**Materials and Methods:**

In this multicentre prospective study, 178 adults with T1D [HCL *n* = 98 (39 males 59 females); MDI + CGM *n* = 80 (30 males 50 females)] who chose to fast in Ramadan of 2024 were assessed for clinical and glycaemic profiles and risk stratified according to the International Diabetes Federation–Diabetes and Ramadan (IDF‐DAR) criteria. The composite endpoint was completing >15 days of fasting and achieving time in range (TIR) ≥70%, with time below range (TBR) <4%.

**Results:**

At baseline, the MDI + CGM group was older than the HCL group (mean ± standard deviation, 29.2 ± 9.6 vs. 26.3 ± 9.4 years) (*p* = 0.049). Half of the HCL participants met the composite endpoint versus only 8% of MDI + CGM users (*p* < 0.001). Similarly, participants in the HCL group were able to complete all possible fasting days more frequently than the MDI + CGM group (*p* < 0.05). The use of HCL was associated with nine‐fold higher odds ratio (OR) (9.4 [95% confidence intervals 3.1–28.4]) of achieving the composite endpoint.

**Conclusions:**

For moderate‐to‐high risk individuals with T1D, HCL substantially improves the safety and feasibility of prolonged daytime fasting during Ramadan, outperforming MDI + CGM in achieving glycaemic and fasting targets. While the results favour HCL, causal inference is still limited, given the lack of randomization and cross‐over design. Concurrently, there is a need to update current recommendations for high‐risk populations and underscore the central role of advanced diabetes technology in Ramadan.

## 1 INTRODUCTION

Fasting forms a core aspect of various religious traditions, and it has also become widely adopted outside these contexts as part of intermittent fasting strategies due to its potential metabolic benefits. Fasting during the month of Ramadan presents unique challenges for people with type 1 diabetes (T1D), who abstain from all oral intake from dawn to dusk for an entire lunar month. Traditional guidelines regard T1D as “high” or “very high risk” for fasting, often discouraging individuals from attempting prolonged fasts due to risks of hypoglycaemia and glycaemic derangements.^1^ Nevertheless, >40% of adult Muslims with T1D choose to fast for spiritual or personal reasons, prompting clinicians to explore safe methods of care during Ramadan.^1^, ^2^, ^3^, ^4^ A more recent guideline issued by the International Diabetes Federation (IDF) and the Diabetes and Ramadan (DAR) International Alliance advocated the use of the IDF‐DAR risk calculator to assess the safety of fasting for individuals with diabetes during Ramadan. It evaluates factors like diabetes type, history of hypoglycaemia or DKA, glycaemic control, and complications to categorize patients into low, moderate or high‐risk groups, guiding fasting recommendations.^4^ Recent publications—including our earlier work—have advocated revisiting these risk classifications to distinguish among varying levels of clinical stability and to leverage modern technologies.^5^, ^6^, ^7^, ^8^

In particular, hybrid closed‐loop (HCL) systems offer the potential to improve T1D management and outcomes in trial and real‐world settings.^8^, ^9^, ^10^, ^11^ HCL's sophisticated algorithms adjust insulin delivery in real‐time based on continuous glucose monitoring (CGM) data. This can lead to a reduction in risks of severe hypoglycaemia and hyperglycaemia previously associated with Ramadan fasting.^8^, ^12^, ^13^, ^14^, ^15^ For individuals at moderate or high risk—commonly labelled “should not fast” in older guidelines—HCL systems may open new possibilities for safe fasting.

We undertook a multicentre prospective real‐world observational study to compare moderate‐to‐high risk adults with T1D who used either HCL or multiple daily injections plus continuous glucose monitoring (MDI + CGM) during Ramadan. Building on prior publications detailing innovative tools for Ramadan,^12^, ^13^, ^14^, ^15^, ^16^, ^17^, ^18^, ^19^, ^20^, ^21^, ^22^ we focused on an integrated composite endpoint that included duration of fasting and glucose metrics. Our goal was to determine whether HCL could allow more individuals with T1D—especially those with historically higher risk scores—to safely complete extended fasts without significant glycaemic deterioration.

## 2 MATERIALS AND METHODS

### 2.1 Study design and participants

We conducted a prospective observational study across three tertiary centres in Saudi Arabia: King Saud University Medical City (KSUMC) in Riyadh, Dallah Hospital in Riyadh and the Royal Commission Hospital in Aljubail. Ethical approval was obtained from King Saud University (E‐24‐8454, dated 18 January 2024). T1D participants on either HCL or MDI + CGM therapy, who were diagnosed for at least 1 year and were planning to fast during Ramadan 2024, were recruited prior to Ramadan 2024. Participants with other forms of diabetes, not fasting or not using CGM were excluded. Written informed consent was obtained. All participants were classified using the IDF‐DAR risk stratification framework (moderate: score 3.5–6; high: score >6).^4^ As previously recommended,^4^ each participant was offered Ramadan‐focused educational discussions, although availability and depth varied among centres. Participants were free to discontinue fasting at any point.

### 2.2 Data collection

Clinical and glycaemic data were recorded for Sha'aban (the month before Ramadan), Ramadan, and Shawwal (the month after Ramadan). Each participant was instructed to log fasting days completed in full, fasting days partially completed and reasons for breaking the fast (e.g., hypoglycaemia, hyperglycaemia, illness, travelling, or menstrual cycle for females, ER visit and DKA). For MDI + CGM users, basal, bolus and correction doses were tracked via questionnaires on Day 15 and Day 30 of Ramadan; CGM data (Freestyle Libre 2) was downloaded from the Libre‐view platform; for HCL users, relevant insulin delivery downloads were extracted from Medtronic CareLink or Tandem Diasend‐Glooko software. In line with recent real‐world Ramadan research (17, 18), time in range (TIR: 70–180 mg/dL), time below range (TBR: <70 mg/dL), time above range (TAR: >180 mg/dL), and glycaemia risk index (GRI) were computed from CGM tracings.^23^ We also documented the incidence of emergency visits, severe hypoglycaemia or diabetic ketoacidosis (DKA).

### 2.3 Composite Endpoint and Other Variables

We introduced a composite endpoint reflecting both fasting duration and glycaemic goals, defined as follows: Fasting >15 days of Ramadan (i.e., at least half the month, the cut‐off has been used in several landmark studies for fasting during Ramadan^24^, ^25^), TIR ≥70% on CGM during Ramadan, and TBR <4% during Ramadan. Participants achieving all three criteria were categorized as having met the composite endpoint. Covariates included age, sex, IDF‐DAR risk stratification, baseline HbA1c, presence of complications, hypoglycaemia awareness, severity and frequency of hypoglycaemia, DKA history, Ramadan counselling, Ramadan experience and type of work intensity.

### 2.4 Statistical analysis

Data were analysed using SPSS v21.0 (IBM, Armonk, NY, USA). Continuous variables are presented as mean ± standard deviation (SD) or mean ± standard error (SE) for ANCOVA‐based estimates, while categorical variables are presented as N (%). Between‐group comparisons used independent t tests (for continuous variables) or chi‐square tests (for categorical variables). Repeated‐measures analysis of covariance (ANCOVA) assessed within‐ and between‐group differences in glycaemic parameters over time, controlling for age. Post‐hoc Bonferroni tests identified specific within‐group changes. Multinomial logistic regression was used to determine variables independently associated with the primary composite outcomes. Odds ratios (ORs) and 95% confidence intervals (CI) are reported. Independent variables included in the model were sex, type of therapy (HCL or MDI + CGM), presence of complications (retinopathy, nephropathy, CVD, PVD), physical activity, history of DKA (in the last 3, 6, 12 months), history of hypoglycaemic awareness, history of hypoglycaemia (≥3 per week, once per week, last 3 months), previous Ramadan experience and Ramadan counselling, adjusted for age and HbA1c. Time in range figures were plotted in MS Excel. Statistical significance was set at *p* < 0.05.

## 3 RESULTS

### 3.1 Baseline characteristics

The HCL group (*n* = 98) included 39 males and 59 females (92 on MiniMed™ 780G and 6 on t:slim X2™ with Control‐IQ). The MDI + CGM group (*n* = 80) included 30 males and 50 females, using multiple daily injections (basal‐bolus regimen) plus FreeStyle Libre 2 CGM system. Demographic and clinical variables are shown in Table 1. Although the MDI + CGM group was older on average (29.2 ± 9.6 vs. 26.3 ± 9.4 years, *p* = 0.049), neither BMI (25.4 ± 5.3 vs. 25.1 ± 5.0 kg/m^2^) nor T1D duration (13.2 ± 9.2 vs. 14.3 ± 9.1 years) differed significantly. Both groups were predominantly high risks per IDF‐DAR scoring [90% (88 participants) in HCL and 96% (77 participants) in MDI+ CGM]. The MDI + CGM cohort had a higher baseline HbA1c (7.6 ± 1.3% vs. 6.9 ± 1.0%, *p* < 0.001) and experienced more frequent weekly hypoglycaemia (≥3 episodes/week; 82.5% vs. 42%, *p* < 0.001). Ramadan‐focused counselling was more common in the MDI + CGM group (82% vs. 57%, *p* < 0.001).

**TABLE 1:** Baseline demographic and clinical characteristics of participants.

Parameters	HCL	MDI + CGM	*p‐*value
*N* (M/F)	98 (39/59)	80 (30/50)	
Age (years)	26.3 ± 9.4	29.2 ± 9.6	0.049
BMI (kg/m^2^)	25.1 ± 5.0	25.4 ± 5.3	0.72
T1DM duration (years)	14.3 ± 9.1	13.2 ± 9.2	0.45
IDF‐DAR Risk (%)			
Moderate	10 (10)	3 (4)	0.15
High	88 (90)	77 (96)	
Complications (%)			
Retinopathy	5 (5)	5 (6)	0.49
Nephropathy	9 (9)	3 (4)	0.13
CVD	0	1 (1)	0.45
PVD	1 (1)	1 (1)	0.70
Reported Work Intensity (Physical labor %)			
High	8 (8)	5 (6)	0.55
Moderate	64 (65)	48 (60)	
Low	26 (27)	27 (34)	
History of DKA during the last (%)			
3 months	5 (5)	4 (5)	0.63
6 months	5 (5)	1 (1)	0.16
12 months	6 (6)	3 (4)	0.36
Severe hypoglycaemia within last 3 months (%)	9 (8)	7 (9)	0.57
Multiple hypoglyacemia episodes (≥ 3/week) (%)	41 (42)	66 (82.5)	<0.001
Hypoglycaemia episode (once/week) (%)	50 (51)	65 (81.3)	<0.001
Ramadan experience (%)			
Negative	9 (9)	14 (18)	0.07
Neutral/Positive	89 (91)	65 (82)	
Counselled on Ramadan fasting (%)	56 (57)	66 (82)	<0.001
HbA1c (%)	6.9 ± 1.0	7.6 ± 1.3	<0.001

### 3.2 Fasting duration and completion rates

Despite greater counselling, participants on MDI + CGM completed fewer overall fasting days compared with HCL users (median 16 vs. 21 days, *p* < 0.001) (Table 2). Most importantly, significantly more HCL users surpassed the 15‐day fasting threshold (90% vs. 76%, *p* = 0.02), and 45% of HCL users completed every possible fasting day, excluding breaks for non‐diabetes reasons, compared with 22% in the MDI + CGM group (*p* = 0.002) (Table 2). The MDI + CGM group reported a higher number of days interrupted by hypoglycaemia (2.5 vs. 1 day, *p* < 0.001) (Table 2). No significant group differences emerged for emergency room visits due to diabetes complications or DKA episodes, which were infrequent (Table 2).

**TABLE 2:** Fasting and glycaemic targets during the month of Ramadan.

	HCL	MDI + CGM	*p*‐value
Fasting days completed#	21 (0–30)	16 (0–30)	<0.001
Goals			
Completed fasting >15 days (%)	88 (90)	61 (76)	0.02
*Completed full fasting days (%)	44 (45)	18 (22)	0.002
Time in range ≥70%	69 (70)	18 (22)	<0.001
Time below range <4%	19 (19)	45 (56)	<0.001
Time above range >25%	38 (39)	59 (74)	<0.001
Broke fasting (days) due to#			
Non‐DM reasons	3 (0–23)	5 (0–14)	0.72
Hypoglycaemia	1 (0–8)	2.5 (0–18)	<0.001
Hyperglycaemia	0 (0–4)	0 (0–14)	0.03
Visited ER due to DM complications (%)	4 (4)	3 (4)	0.61
DKA episode (%)	2 (2)	0	0.30

*Note*: Data presented as absolute numbers (%) for categorical variables; *defined as total successful fasting days out of potential 30 days (30 – non‐fasting days for non‐DM reasons); # presented as median (min‐max).

### 3.3 Glycaemic metrics and composite endpoint

**FIGURE 1:**
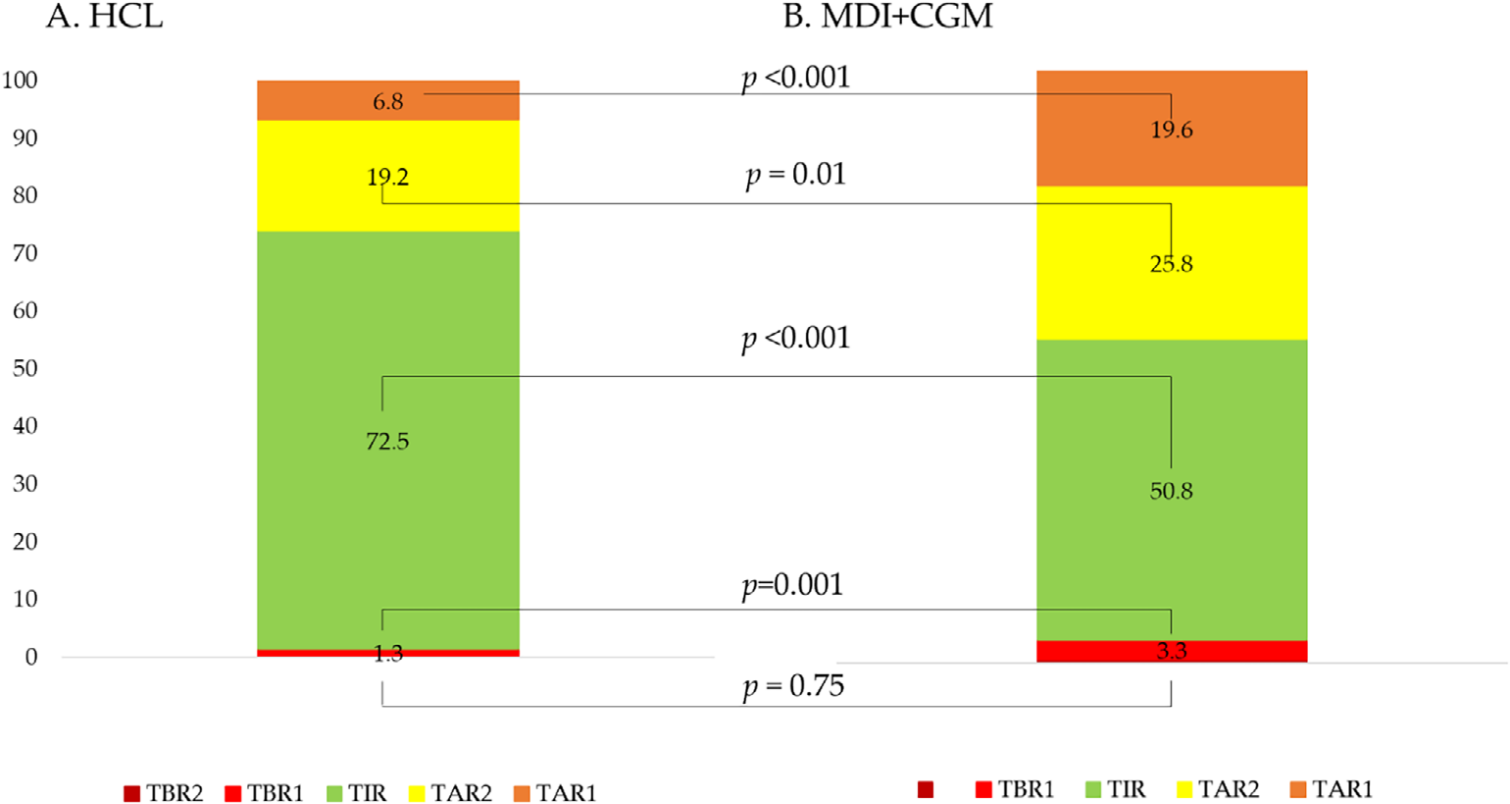
Glycaemic metrics for HCL versus MDI + CGM groups during Ramadan.

Within‐ (group × time) and between‐group comparisons over time are presented in Table 3. Nearly all CGM metrics (Figure 1, Table 3) were favourable for HCL vs. MDI + CGM groups before, during and after Ramadan. Apart from the proportion of very low readings (<54 mg/dL), MDI + CGM group showed significant variation in glycaemic metrics during Ramadan. Significant shifts in sensor glucose and GMI emerged only within the MDI + CGM group. Meanwhile, neither group experienced significant changes in the coefficient of variation (CV) over time, though the MDI + CGM group's CV was notably higher than that of the HCL group when compared across groups.

**TABLE 3:** Insulin dosages, glycaemic parameters and TIR before, during and after Ramadan.

Parameters	HCL			MDI + CGM			Between grou*p*	Group* time
	Before	During	After	Before	During	After		
***Time in range***								
Very high (>250 mg/dL)	5.9 ± 1.0	6.8 ± 1.1	6.0 ± 1.1	14.6 ± 1.1	19.6 ± 1.2^A^	15.4 ± 1.2^B^	<0.001	<0.001
High (181‐250 mg/dl)	18.8 ± 0.7	19.2 ± 0.7	18.8 ± 0.8	22.8 ± 0.8	25.8 ± 0.8^A^	23.0 ± 0.9^B^	<0.001	0.012
Target range (70‐180 mg/dl)	73.3 ± 1.3	72.5 ± 1.4	73.6 ± 1.3	57.2 ± 1.5	50.8 ± 1.5^A^	56.3 ± 1.4^B^	<0.001	<0.001
Low (54‐69 mg/dL)	1.4 ± 0.3	1.3 ± 0.2	1.3 ± 0.2	4.6 ± 0.3	3.3 ± 0.2^A^	4.5 ± 0.3^B^	<0.001	0.001
Very low (<54 mg/dl)	0.3 ± 0.1	0.2 ± 0.1	0.3 ± 0.1	0.8 ± 0.1	0.6 ± 0.1	0.8 ± 0.1	<0.001	0.747
Basal insulin dose/day	17.5 ± 1.1	17.9 ± 1.0		24.6 ± 1.1	22.6 ± 1.1^A^		<0.001	0.011
Average bolus/day	19.6 ± 1.5	19.4 ± 1.3		29.7 ± 1.6	26.0 ± 1.4^A^		<0.001	0.010
Average Auto‐Correction (U/day)	6.0 ± 0.4	6.4 ± 0.4						
GRI	29.0 ± 1.8	29.9 ± 1.9	28.4 ± 1.7	55.0 ± 2.0	61.7 ± 2.1^A^	56.3 ± 1.9^B^	<0.001	0.007
Sensor glucose mg/dl	151.6 ± 2.6	154.5 ± 2.8	160.7 ± 8.6	168.5 ± 2.9	183.1 ± 3.1	173.8 ± 9.5	0.001	0.30
GMI	6.9 ± 0.1	7.2 ± 0.2	7.2 ± 0.2	7.3 ± 0.1	7.7 ± 0.2	7.4 ± 0.3	0.12	0.74
CV	33.4 ± 3.0	32.5 ± 0.6	32.3 ± 3.0	43.1 ± 3.3	37.3 ± 0.7	43.5 ± 3.3	<0.001	0.46

*Note*: Presented as estimated means ± SE; Group denotes between‐group *p*‐value; Group*time denotes *p*‐value interaction; both *p*‐values adjusted for age and HbA1c. Superscripts ^A^ denote significance compared with before Ramadan and ^B^ for during Ramadan; Significant at *p* < 0.01.

HCL users also maintained steadier glycaemia risk index (GRI) across Sha'aban, Ramadan and Shawwal (26.7 ± 1.8, 27.3 ± 2.0 and 25.8 ± 1.8, respectively), whereas GRI was higher and fluctuated sharply in MDI + CGM participants (57.4 ± 2.0, 64.8 ± 2.2 and 59.3 ± 2.1, *p* = 0.001 for HCL vs. MDI + CGM) (Table 3).

**FIGURE 2:**
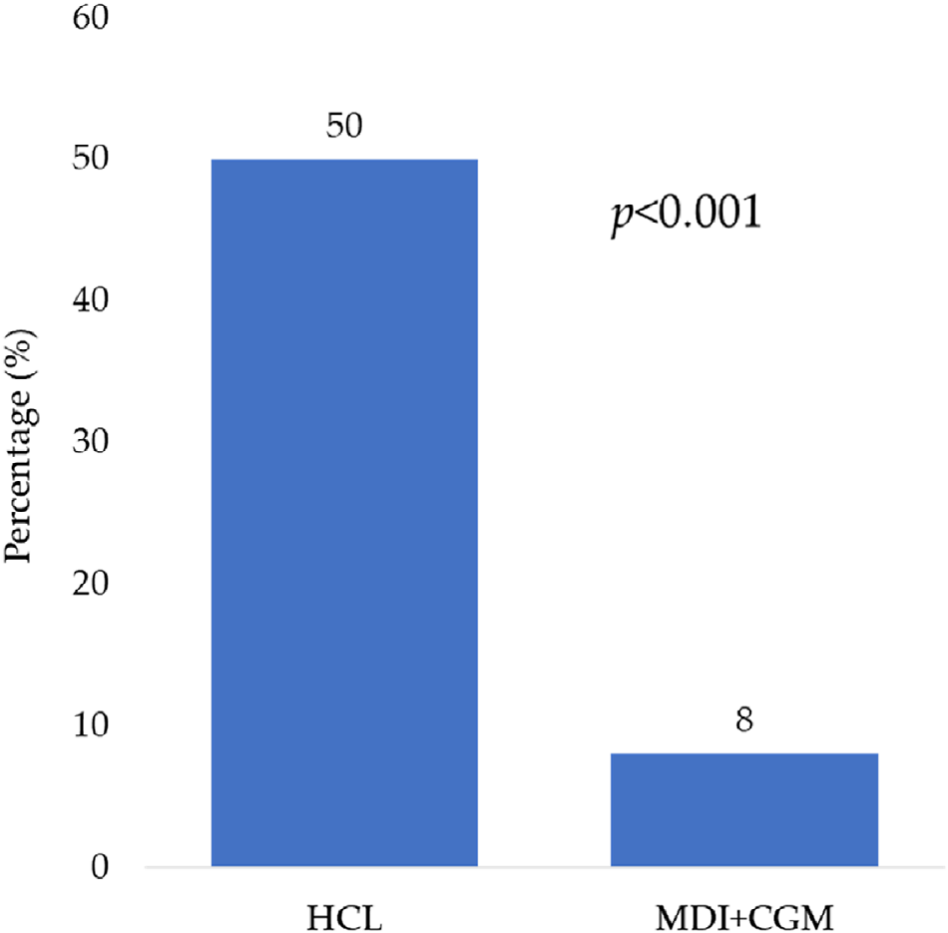
Proportion (%) of participants achieving composite endpoint (completed fasting >15 days, TIR ≥70% and TBR <4% during Ramadan) in HCL versus MDI + CGM.

Time‐series comparisons of insulin dosing revealed that MDI + CGM participants significantly lowered both basal (−1.9 units/day) and bolus doses (−3.8 units/day) during Ramadan, presumably to mitigate hypoglycaemia risks, whereas the HCL group showed stable insulin delivery over time (Table 2). Compared with MDI + CGM, the HCL group achieved markedly better TIR ≥70% (70% vs. 22%, *p* < 0.001) and less TBR ≥4% (19% vs. 56%, *p* < 0.001). The proportion meeting our composite endpoint (fasting >15 days, TIR ≥70%, TBR <4%) was 50% in the HCL group versus 8% in the MDI + CGM group (*p* < 0.001) (Figure 2). As shown in Table 4, use of HCL was the strongest predictor of achieving the composite endpoint (OR 9.4, 95% CI 3.1–28.4, *p* < 0.001). Absence of DKA in the last 12 months and infrequent hypoglycaemia (<1/week) also contributed significantly to reaching the endpoint (*p* < 0.05 for both). Ramadan counselling did not reach statistical significance (OR 4.6, 95% CI 0.85–25.4, *p* = 0.08), although prior studies have shown that structured education programmes can improve safety and confidence.^17^

**TABLE 4:** Multinomial Logistic Regression: Predictors of the Composite Endpoint.

Parameter	Odds‐Ratio	95% CI	*p*‐value
Treatment			
MDI	1.0		
Pump	7.1	2.3–21.9	<0.001
Work intensity (Physical Labor)			
Low	1.0		
Moderate	4.2	1.3–12.7	0.01
High	0.67	0.10–4.2.3	0.67
History of DKA (w/in 12 mos)			
Yes	1.0		
No	10.3	1.4–76.8	0.02
Hypoglycaemia once per week			
Yes	1.0		
No	0.37	0.2–0.91	0.03

*Note*: All Exp (β) coefficients (odds‐ratios) were adjusted for age and HbA1c. Only significant/borderline significant parameters were presented. Significant at *p* < 0.05.

## 4 DISCUSSION

We demonstrated that, in moderate‐ to high‐risk individuals with T1D, HCL systems can deliver safe and effective Ramadan management compared with MDI + CGM, yielding significant advantages in glycaemic metrics—including GMI, GRI, and our composite endpoint. These findings align with prior data, yet our high proportion of high‐risk participants (92%) differentiates this study from earlier work.^8^, ^13^, ^14^ While older guidelines classified such cohorts as “should not fast,” emerging evidence—including our own—indicates that real‐world adoption of HCL can substantially reduce glycaemic extremes.^5^ Indeed, half of HCL users here achieved an ambitious composite endpoint spanning both fasting duration and glycaemic control, paralleling other reports that underscore how pairing diabetes technology with structured education can empower safe fasting.^17^ This mounting body of evidence supports moving away from blanket “no fasting” recommendations for high‐risk T1D, favouring more nuanced risk stratification.^4^, ^5^, ^7^, ^26^

Although over two‐thirds of participants in both groups fasted beyond 15 days, 45% of HCL users completed the entire month—versus only 22% on MDI + CGM—demonstrating HCL's real‐world impact on limiting abrupt hypoglycaemia or hyperglycaemia, primary triggers for broken fasts. Nevertheless, a sizable subgroup of HCL users could not maintain fasting for all of Ramadan. Whilst non‐diabetes issues accounted for most of the contributing factors, hypoglycaemia‐related reasons for cessation of fasts were still evident in HCL users, albeit at a significantly reduced level compared with MDI + CGM. Previously, the mini‐dose glucagon as an adjunct to MDI + CGM for preventing fasting‐related hypoglycaemia was explored.^6^ Future research on its utility in T1DM and combining HCL with mini‐dose glucagon may offer further strategies for those at elevated hypoglycaemia risk.

It is worth mentioning that safer fasting in those without a history of DKA was a significant predictor for achieving the composite endpoint. The IDF‐DAR risk calculator recognizes the significance of a recent history of DKA by assigning higher points for DKA within the last 3 months than occurrences within the last six or nine months.^4^ A recent DKA event often indicates challenges in management and coping, whether due to negligence, inadequate understanding or failure to increase insulin when necessary (e.g., during illness). These factors place the patient at a higher risk. Safe fasting during Ramadan requires patients to be vigilant, monitor their blood glucose levels closely, recognize symptoms, adjust their insulin doses accordingly, and make informed decisions about breaking the fast if needed. Patients who have experienced a recent DKA event may lack the necessary skills for safe fasting or may not be fit to fast. Although these individuals have the option not to fast, many still choose to do so for various personal reasons. As healthcare workers, these findings underscore the importance of paying closer attention to patients with a recent history of DKA, as they may require additional support and guidance.

Meanwhile, the glycaemia risk index (GRI),^27^ used as a composite measure of glycaemic control, stayed stable in the HCL group but fluctuated significantly in MDI + CGM users, mirroring shifts in average daily bolus. This rise appears to be related to common practices during Ramadan, where patients on MDI often reduce their insulin doses to avoid hypoglycaemia and the need to break their fast and/or underestimate their insulin requirement with meals^7^ —decisions that may not always align with medical recommendations. As shown in the glucose metrics, there was a significant increase in both TAR 1 and 2 during Ramadan for the MDI group. This indicates a greater proportion of time spent in hyperglycaemia compared with the surrounding months, which in turn contributed to the elevated GRI score. Ironically, despite these self‐initiated dose adjustments and more pre‐Ramadan counselling in MDI + CGM users, hypoglycaemic events remained higher in that group. By contrast, HCL users adjusted insulin less frequently yet realized better glycaemic outcomes. Future work should integrate quality‐of‐life metrics, examine cost‐effectiveness, and explore adjunctive therapies—such as mini‐dose glucagon or dual‐hormone systems—to refine safe fasting protocols for people with T1D across diverse cultural and socioeconomic contexts.

We acknowledge several limitations. First, we did not measure formal quality‐of‐life or psychosocial indicators, though other investigations suggest that HCL use generally confers benefits in these domains.^28^ Second, this was a real‐world study without laboratory‐based HbA1c evaluations, but numerous trials have demonstrated strong consistency between CGM‐based metrics and lab measures. Although the design does not fully isolate the effect of HCL from potential confounders, the consistency of our findings with previously reported benefits of HCL in both randomized and observational studies strengthens the clinical relevance of our results. Nevertheless, we also acknowledge the absence of randomization or a cross‐over design. While the results favour HCL, causal inference is limited. The HCL group had significantly lower HbA1c, was younger and experienced fewer hypoglycaemic episodes at baseline, suggesting they may have already been better controlled or more engaged in diabetes management. Third, our MDI + CGM group tended to be modestly older than HCL users, an inherent demographic difference in real‐world settings that was not fully controllable. Nonetheless, our multicentre real‐world approach and relatively large high‐risk sample for a Ramadan study strengthens the generalizability of our results. Lastly, all CGM metrics were extracted from different CGM platforms and, hence, limit the validity of comparisons.

In summary, for moderate‐ to high‐risk T1D adults, HCL systems appear to markedly improve both the safety and effectiveness of extended daytime fasting during Ramadan. By extending time‐in‐range, reducing hypoglycaemia and enabling completion of more fasting days, HCL users surpassed MDI + CGM users in a composite endpoint encompassing fasting duration and glycaemic goals. These data align with emerging literature advocating more flexible, technology‐driven approaches for Ramadan. We propose that current “no fasting” guidance for high‐risk T1D populations may need to be revisited, acknowledging the real‐world benefits of advanced systems. These findings may have broader applicability beyond Ramadan, including other religious fasts and intermittent fasting practices. HCL systems offer allows for automated basal insulin delivery adjustments helping to mitigate hypoglycaemia and glycaemic variability. Their use can support user autonomy in adjusting meal timing without compromising glycaemic control and therefore achieve safe fasting across diverse cultural and clinical contexts. To conclude, for moderate‐ to high‐risk T1D adults, HCL systems are associated with improvements in safety and effectiveness of extended daytime fasting during Ramadan.

## FUNDING INFORMATION

This study did not receive any funding. SH is supported by the UK Research and Innovation (UKRI) / Medical Research Council (MRC) award (MR/W030004/1).

## CONFLICT OF INTEREST STATEMENT

AMA: has served on an advisory panel for Medtronic, Novonordisk, Eli Lilly, Vital Air and Sanofi and has received honoraria for speaking from AstraZeneca, Eli Lilly, Medtronic, Novo Nordisk and Sanofi. SH has served on the advisory board for Tandem, Dexcom, Medtronic, Sanofi, Vertex; undertaken non‐promotional educational and/or consultancy work for Abbott UK, Insulet, Dexcom and Roche; SH's institution has received research grant support from Abbott and Insulet. JK has served on the advisory board for AstraZeneca, Boehringer Ingelheim and Sanofi. All other authors declare no conflict of interest.

## PEER REVIEW

The peer review history for this article is available at https://www.webofscience.com/api/gateway/wos/peer‐review/10.1111/dom.16573.

## Data Availability

Data supporting the findings are available within the article. Further details are available upon request.
